# A statistical approach for identifying differential distributions in single-cell RNA-seq experiments

**DOI:** 10.1186/s13059-016-1077-y

**Published:** 2016-10-25

**Authors:** Keegan D. Korthauer, Li-Fang Chu, Michael A. Newton, Yuan Li, James Thomson, Ron Stewart, Christina Kendziorski

**Affiliations:** 1 Department of Biostatistics and Computational Biology, Dana-Farber Cancer Institute, Boston, 02215 MA USA; 2 Department of Biostatistics, Harvard T. H. Chan School of Public Health, Boston, 02115 MA USA; 3 Morgridge Institute for Research, University of Wisconsin, Madison, 53706 WI USA; 4 Department of Biostatistics, University of Wisconsin, Madison, 53706 WI USA; 5 Department of Statistics, University of Wisconsin, Madison, 53706 WI USA; 6 Department of Cell and Regenerative Biology, University of Wisconsin, Madison, 53706 WI USA; 7 Department of Molecular, Cellular, and Developmental Biology, University of California, Santa Barbara, 93106 CA USA

**Keywords:** Single-cell RNA-seq, Differential expression, Cellular heterogeneity, Mixture modeling

## Abstract

The ability to quantify cellular heterogeneity is a major advantage of single-cell technologies. However, statistical methods often treat cellular heterogeneity as a nuisance. We present a novel method to characterize differences in expression in the presence of distinct expression states within and among biological conditions. We demonstrate that this framework can detect differential expression patterns under a wide range of settings. Compared to existing approaches, this method has higher power to detect subtle differences in gene expression distributions that are more complex than a mean shift, and can characterize those differences. The freely available R package scDD implements the approach.

**Electronic supplementary material:**

The online version of this article (doi:10.1186/s13059-016-1077-y) contains supplementary material, which is available to authorized users.

## Background

**Fig. 1:**
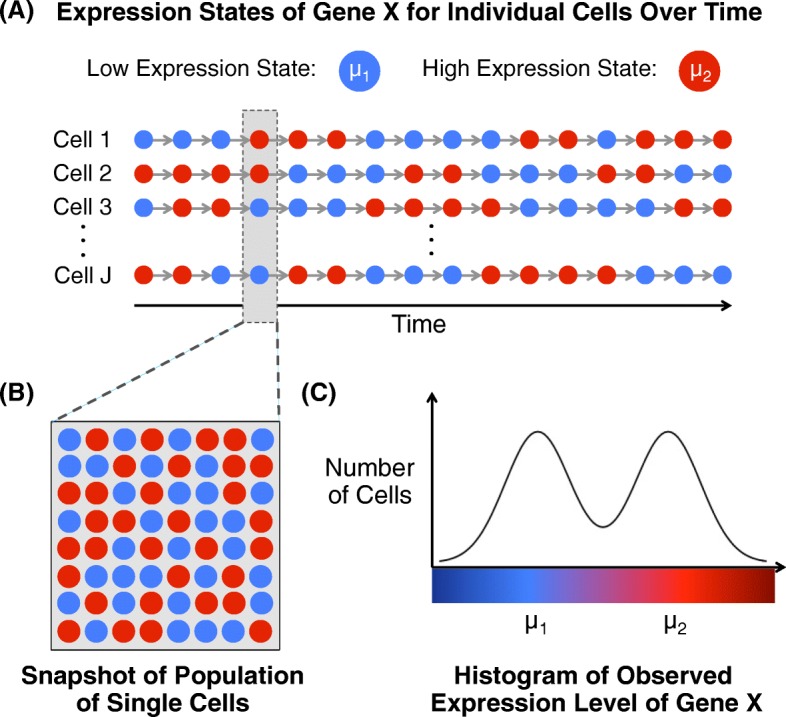
Schematic of the presence of two cell states within a cell population that can lead to bimodal expression distributions. **a** Time series of the underlying expression state of gene X in a population of unsynchronized single cells, which switches back and forth between a low and high state with means *μ*_1_ and *μ*_2_, respectively. The color of cells at each time point corresponds to the underlying expression state. **b** Population of individual cells shaded by expression state of gene X at a snapshot in time. **c** Histogram of the observed expression level of gene X for the cell population in (**b**)

Coordinated gene expression is fundamental to an organism’s development and maintenance, and aberrations are common in disease. Consequently, experiments to measure expression on a genome-wide scale are pervasive. The most common experiment involves the quantification of mRNA transcript abundance averaged over a population of thousands or millions of cells. These so-called traditional, or bulk, RNA-seq experiments have proven useful in a multitude of studies. However, because bulk RNA-seq does not provide a measure of cell-specific expression, many important signals go unobserved. A gene that appears to be expressed at a relatively constant level in a bulk RNA-seq experiment, for example, may actually be expressed in sub-groups of cells at levels that vary substantially (see Fig. 1).

Single-cell RNA-seq (scRNA-seq) facilitates the measurement of genome-wide mRNA abundance in individual cells, and as a result, provides the opportunity to study the extent of gene-specific expression heterogeneity within a biological condition, and the impact of changes across conditions. Doing so is required for discovering novel cell types [1, 2], for elucidating how gene expression changes contribute to development [3–5], for understanding the role of cell heterogeneity on the immune response [6, 7] and cancer progression [6, 8–10], and for predicting the response to chemotherapeutic agents [11–13]. Unfortunately, the statistical methods available for characterizing gene-specific expression within a condition and for identifying differences across conditions in scRNA-seq are greatly limited, largely because they do not fully accommodate the cellular heterogeneity that is prevalent in single-cell data.

To identify genes with expression that varies across biological conditions in an scRNA-seq experiment, a number of early studies used methods from bulk RNA-seq [4, 10, 12, 14, 15]. In general, the methods assume that each gene has a latent level of expression within a biological condition, and that measurements fluctuate around that level due to biological and technical sources of variability. In other words, they assume that gene-specific expression is well characterized by a unimodal distribution within a condition. Further, tests for differences in expression to identify so-called differentially expressed (DE) genes amount to tests for shifts in the unimodal distributions across conditions. A major drawback of these approaches in the single-cell setting is that, due to both biological and technical cell-to-cell variability, there is often an abundance of cells for which a given gene’s expression is unobserved [7, 16, 17] and, consequently, unimodal distributions are insufficient.

**Fig. 2:**
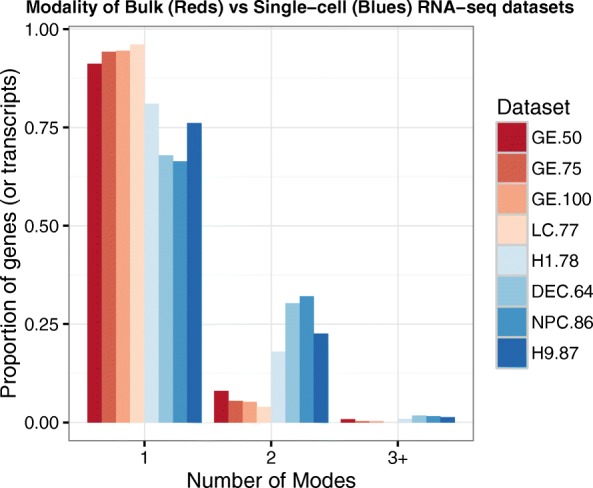
Comparison of modality in bulk versus single cells. Bar plot of the proportion of genes (or transcripts) in each dataset where the log-transformed nonzero expression measurements are best fit by a 1, 2, or 3 + mode normal mixture model (where 3+ denotes 3 or more). Modality is determined using a Bayesian information selection criterion with filtering (see “Partition estimation”). *Red shades* denote bulk RNA-seq datasets, and *blue shades* denote single-cell datasets. The number following each dataset label indicates the number of samples present (e.g., *GE.50* is a bulk dataset with 50 samples). Datasets *GE.50*, *GE.75*, and *GE.100* are constructed by randomly sampling 50, 75, and 100 samples from GEUVADIS [56]. Dataset *LC* consists of 77 normal samples from the TCGA lung adenocarcinoma study [57]. For details of the single-cell datasets, see “Methods”

To address this, a number of statistical methods have been developed recently to accommodate bimodality in scRNA-seq data [17, 18]. In these mixture-model based approaches, one component distribution accommodates unobserved, or dropout, measurements (which include zero and, optionally, thresholded low-magnitude observations) and a second unimodal component describes gene expression in cells where expression is observed. Although these approaches provide an advance over unimodal models used in bulk, they are insufficient for characterizing multi-modal expression data, which is common in scRNA-seq experiments (see Fig. 2).

Specifically, a number of studies have shown that many types of heterogeneity can give rise to multiple expression modes within a given gene [19–23]. For example, there are often multiple states among expressed genes [19, 20, 22] (a schematic is shown in Fig. 1). The transition between cell states may be primarily stochastic in nature and result from expression bursts [24, 25], or result from positive feedback signals [19, 23, 26]. Beyond the existence of multiple stable states, multiple modes in the distribution of expression levels in a population of cells may also arise when the gene is either oscillatory and unsynchronized, or oscillatory with cellular heterogeneity in frequency, phase, and amplitude [21, 23].

**Fig. 3:**
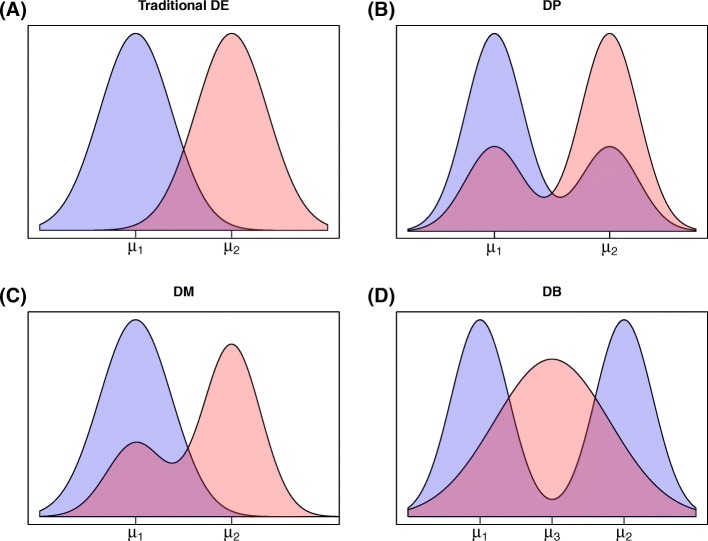
Diagram of plausible differential distribution patterns (smoothed density histograms), including **a** traditional differential expression (DE), **b** differential proportion of cells within each component (DP), **c** differential modality (DM), and **d** both differential modality and different component means within each condition (DB). *DB* both differential modality and different component means, *DE* differential expression, *DM* differential modality, *DP* differential proportion

Figure 3 illustrates common multi-modal distributions within and across biological conditions. When the overall mean expression level for a given gene is shifted across conditions, then bulk methods, or recent methods for scRNA-seq [17, 18, 27, 28], may be able to identify the gene as showing some change. However, as we show here, they would be relatively underpowered to do so, and they would be unable to characterize the change, which is often of interest in an scRNA-seq experiment. For example, the gene in Fig. 3 c shows a differential number of modes (DM), while the gene in Fig. 3 b shows a differential proportion (DP) of cells at each expression level across conditions. Differentiating between DM and DP is important since the former suggests the presence of a distinct cell type in one condition, but not the other, while the latter suggests a change in splicing patterns among individual cells [7] or cell-specific responses to signaling [29].

Here we develop a Bayesian modeling framework, scDD, to facilitate the characterization of expression within a biological condition, and to identify genes with differential distributions (DDs) across conditions in an scRNA-seq experiment. A DD gene may be classified as DE, DM, DP, or both DM and differential means of expression states (abbreviated DB). Figure 3 provides an overview of each pattern. Simulation studies suggest that the approach provides improved power and precision for identifying differentially distributed genes. Additional advantages are demonstrated in a case study of human embryonic stem cells (hESCs).

## Results and discussion

### Human embryonic stem cell data

**Fig. 4:**
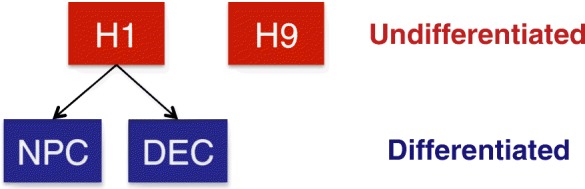
Relationship of cell types used in hESC case study. *H1* and *H9* are undifferentiated hESC lines. *NPC* (neuronal progenitor cells) and *DEC* (definitive endoderm cells) are differentiated cell types derived from *H1*. *DEC* definitive endoderm cell, *NPC* neuronal progenitor cell

scRNA-seq data were generated in the James Thomson Lab at the Morgridge Institute for Research (see “Methods” and [30] for details). Here we analyze data from two undifferentiated hESC lines: the male H1 line (78 cells) and the female H9 line (87 cells). In addition, we include data from two differentiated cell types that are both derived from H1: definitive endoderm cells (DECs, 64 cells) and neuronal progenitor cells (NPCs, 86 cells). The relationship between these four cell types is summarized by the diagram in Fig. 4. As discussed in the case study results, it is of interest to characterize the differences in distributions of gene expression among these four cell types to gain insight into the genes that regulate the differentiation process.

### Publicly available human myoblast and mouse embryonic stem cell data

We also apply our method to two publicly available scRNA-seq datasets to determine which genes are differentially distributed following stimulation or inhibition of differentiation via a specialized growth medium. Using data from [31], we compare gene expression of human myoblast cells cultured in standard growth medium (T0, 96 cells) with those treated with differentiation-inducing medium for 72 hours (T72, 84 cells). Additionally, we use data from [32] to compare the gene expression of mouse embryonic stem cells (mESCs) cultured in standard medium (Serum + LIF, 93 cells) with those cultured on differentiation-inhibiting medium (2i + LIF, 94 cells).

### Simulated data

We evaluate model performance using log-transformed count data simulated from mixtures of negative binomial distributions. The analysis of log-transformed counts from bulk RNA-seq has been shown to perform as well as utilizing count-based modeling assumptions [33, 34]. Recent scRNA-seq analyses have also assumed the normality of log-transformed nonzero measurements [7, 18]. For each simulated dataset, 10,000 genes were simulated for two conditions with four different sample size settings (50, 75, 100, and 500 cells in each condition). The majority of the genes (8000) were simulated out of the same model in each condition, and the other 2000 represent genes with the four types of DD outlined in Fig. 3. The 2000 DD genes were split equally into the following four categories:

- DE: single component with a different mean in each condition
- DP: two components in each condition with equal component means across conditions; the proportion in the low mode is 0.33 for condition 1 and 0.66 for condition 2
- DM: single component in condition 1; two components in condition 2 with one overlapping component. Half of the condition 2 cells belong to each mode
- DB: single component in condition 1; two components in condition 2 with no overlapping components. The mean of condition 1 is half-way between the means in condition 2. Half of the cells in condition 2 belong to each mode

Here a component represents the distribution of expression values at a particular expression level (or mode), and different biological groups of interest are referred to as conditions. Of the 8000 null genes, 4000 were generated from a single negative binomial component (EE, or equivalent expression) and the other 4000 from a two-component negative binomial mixture (EP, or equivalent proportions of cells belonging to each component). The parameters of the negative binomial distributions for the unimodal genes were chosen to be representative of the observed means and variances in the H1 dataset. Fold-changes for DE genes were chosen to be representative of those observed in the H1 and DEC comparison. Distances between (log-scale) component means *Δ*_*μ*_ *σ* (referred to as component mean distance) in the multi-modal genes were varied, with an equal proportion of genes at each setting of *Δ*_*μ*_∈{2,3,4,5,6}, where *σ* is the within-component standard deviation on the log-scale (simulated to be common across components for a given gene and condition). More details are provided in “Methods”.

### The scDD modeling framework

Let *Y*_*g*_=(*y*_*g*1_,…,*y*_*gJ*_) be the log-transformed nonzero expression measurements of gene *g* in a collection of *J* cells from two biological conditions. We assume that measurements have been normalized to adjust for technical sources of variation including amplification bias and sequencing depth. Under the null hypothesis of equivalent distributions (i.e., no dependence on condition), we let *Y*_*g*_ be modeled by a conjugate Dirichlet process mixture (DPM) of normals (see “Methods” for more details). Gene *g* may also have expression measurements of zero in some cells; these are modeled as a separate distributional component (see “Differential proportion of zeroes” for more details).

Ultimately, we would like to calculate a Bayes factor for the evidence that the data arises from two independent condition-specific models (DDs) versus one overall model that ignores condition (equivalent distributions or EDs). Let $\mathcal {M}_{\text {DD}}$ denote the DD hypothesis, and $\mathcal {M}_{\text {ED}}$ denote the equivalent distribution hypothesis. A Bayes factor in this context for gene *g* would be:

$$ \operatorname{BF}_{g} = \frac{f(Y_{g} | \mathcal{M}_{\text{DD}})}{f(Y_{g} | \mathcal{M}_{\text{ED}})} $$

where $f(Y_{g} | \mathcal {M})$ denotes the predictive distribution of the observations from gene *g* under the given hypothesis. In general, there is no analytical solution for this distribution under the DPM model framework. However, under the product partition model (PPM) formulation (see “Methods” for more details), we can get a closed form solution for $f(Y_{g}, Z_{g} | \mathcal {M})$, where *Z*_*g*_ represents a partition (or clustering) of samples to mixture components. As the partition *Z*_*g*_ cannot be integrated out, we introduce an approximate Bayes factor score:

$$\begin{aligned} \operatorname{Score}_{g} &= \log \left(\frac{f(Y_{g}, Z_{g} | \mathcal{M}_{\text{DD}})}{f(Y_{g}, Z_{g} | \mathcal{M}_{\text{ED}})}\right)\\ &= \log\left(\frac{f_{C1}(Y_{g}^{C1}, Z_{g}^{C1}) f_{C1}(Y_{g}^{C2}, Z_{g}^{C2})}{f_{C1,C2}(Y_{g}, Z_{g})} \right) \end{aligned} $$

where *C*1 and *C*2 denote conditions 1 and 2, respectively, and the score is evaluated at the partition estimate $\hat {Z_{g}}$. A high value of this score presents evidence that a given gene is differentially distributed. The significance of the score is assessed via a permutation test. Specifically, condition labels are permuted and partition estimates are obtained within the new conditions. For each permuted dataset, the Bayes factor score is calculated; the default in scDD is 1000 permutations. For each gene, an empirical *p* value is calculated, and the false discovery rate (FDR) is controlled for a given target value using the method of [35].

If covariates are available, instead of permuting the observed values, the relationship between the clustering and covariates can be preserved by permuting the residuals of a linear model that includes the covariate and using the fitted values [36]. As pointed out by [18], the cellular detection rate is a potential confounder variable, so the permutation procedure in the case studies is adjusted in this manner. If other known confounders exist and are measured, these can also be incorporated in the same manner. Note that while this procedure adjusts for covariates that affect mean expression levels, it does not adjust for covariate-specific effects on variance. The sensitivity of the approach to various levels of nonlinear confounding effects is evaluated in a simulation study presented in Additional file 1: Section 2.3.

### Classification of significant DD genes

For genes that are identified as DD by the Bayes factor score, of interest is classifying them into four categories that represent the distinct DD patterns shown in Fig. 3. To classify the DD genes into these patterns (DE, DM, DP, and DB), scDD utilizes the conditional posterior distribution of the component-specific mean parameters given in Eq. 6 (see “Methods”). Posterior sampling is carried out to investigate the overlap of components across conditions. Let *c*_1_ be the number of components in condition 1, *c*_2_ the number of components in condition 2, and *c*_OA_ the number of components overall (when pooling conditions 1 and 2). Only components containing at least three cells are considered to minimize the impact of outlier cells. Note that for interpretability, a DD gene must satisfy: *c*_1_+*c*_2_≥*c*_OA_≥ min(*c*_1_,*c*_2_). These bounds on the overall number of components represent the two extreme cases: condition 1 does not overlap with condition 2 at all, versus one condition completely overlaps with the other. Any cases outside of these boundaries are not readily interpretable in this context. The actions to take for all other possible combinations of *c*_1_, *c*_2_, and *c*_OA_ are detailed in “Methods”.

### Differential proportion of zeroes

For those genes that do not show DDs in the nonzero values, scDD allows a user to evaluate whether the proportion of zeroes differs significantly between the two conditions. This evaluation is carried out using logistic regression adjusted for the proportion of genes detected in each cell as in [18]. Genes with a *χ* ^2^ test *p* value of less than 0.025 (after adjustment for multiple comparisons using the method of [35]) are considered to have a differential proportion of zeroes (DZ).

### Simulation study

A simulation study was conducted to assess the performance of scDD in identifying DD genes, and to classify them as DE, DP, DM, or DB. Model performance on the simulated data was assessed based on (1) the ability to estimate the correct number of components, (2) the ability to detect significantly DD genes, and (3) the ability to classify DD genes into their correct categories. These three criteria are explored in the next three sections, respectively. Existing methods for DE analysis are also evaluated for the second criterion.

#### Estimation of the number of components

We first examine the ability of scDD to detect the correct number of components. Table 1 displays the proportion of bimodal and unimodal simulated genes where the correct number of components was identified. For bimodal genes, results are stratified by component mean distance. It is clear that the ability of the algorithm to identify the correct number of components in bimodal genes improves as the component mean distance or sample size increases. The results for unimodal genes are not as sensitive to sample size; however, the proportion of genes identified as bimodal increases slightly with more samples. We conclude that the partition estimate is able to detect reliably the true number of components for reasonable sample and effect sizes.

**Table 1:** Rate of detection of correct number of components in simulated data

	Bimodal					Unimodal
Sample	component mean distance *Δ*_*μ*_					
size	2	3	4	5	6	
50	0.056	0.196	0.579	0.848	0.922	0.907
75	0.052	0.252	0.719	0.917	0.957	0.908
100	0.050	0.302	0.811	0.950	0.974	0.905
500	0.073	0.417	0.959	0.995	0.991	0.884

Average proportion of simulated bimodal and unimodal genes where the correct number of components was identified, averaged over gene category and condition. Averages are calculated over 20 replications. Standard errors were <0.025 (not shown)

#### Detection of DD genes

Next, we examine the ability of scDD to identify the non-null genes as significantly DD, and compare it to existing methods, SCDE [17] and MAST [18]. For each method, the target FDR was set at 5 % (see “Methods” for details). The power to detect each gene pattern as DD for all three methods is shown in Table 2. Note that the calculations here are taken before the classification step for scDD, so power is defined as the proportion of genes from each simulated category that are detected as DD. In general, the power to detect DD genes improves with increased sample size for all three methods. Our approach has comparable power to SCDE and MAST for DE and DP genes, but higher overall power to detect DM and DB genes. Interestingly, SCDE has very low power to detect DP genes, whereas MAST shows very low power to detect DB genes. We note that SCDE and MAST do not aim to detect genes with no change in the overall mean level in expressed cells (as in the case of DB genes), so it is expected that scDD will outperform other methods at detecting genes in this category.

**Table 2:** Power to detect DD genes in simulated data

		True gene category				
Sample size	Method	DE	DP	DM	DB	Overall (FDR)
50	scDD	0.893	0.418*	0.898*	0.572*	0.695* (0.029)
	SCDE	0.872	0.026	0.817	0.260	0.494 (0.004)
	MAST	0.908*	0.400	0.871	0.019	0.550 (0.026)
75	scDD	0.951	0.590	0.960*	0.668*	0.792* (0.031)
	SCDE	0.948	0.070	0.903	0.387	0.577 (0.003)
	MAST	0.956*	0.633*	0.943	0.036	0.642 (0.022)
100	scDD	0.972	0.717	0.982*	0.727*	0.850* (0.033)
	SCDE	0.975	0.125	0.946	0.478	0.631 (0.003)
	MAST	0.977*	0.752*	0.970	0.045	0.686 (0.022)
500	scDD	1.000*	0.983	1.000*	0.905*	0.972* (0.035)
	SCDE	1.000*	0.855	0.998	0.787	0.910 (0.004)
	MAST	1.000*	0.993*	1.000*	0.170	0.791 (0.022)

Average power to detect simulated DD genes by true category. Averages are calculated over 20 replications. Standard errors were <0.025 (not shown)

*DB* both differential modality and different component means, *DD* differential distribution, *DE* differential expression, *DM* differential modality, *DP* differential proportion, *FDR* false discovery rate. Values followed by * designate which method(s) achieved the highest power to detect DD genes from each particular gene category (as well as overall) for each sample sample size setting

#### Classification of DD genes

Next, we examine the ability of scDD to classify each DD gene into its corresponding category. Table 3 shows the correct classification rate in each category for DD genes that were correctly identified during the detection step (calculated as the proportion of true positive genes detected as DD for a given category that were classified into the correct category). The classification rates do not depend strongly on sample size, with the exception of DP, which decreases with increasing sample size. This decrease results from an increase in the DD detection rate of DP genes with small component mean distance, which have a lower correct classification rate (as shown below).

**Table 3:** Correct classification rate in simulated data

	Gene category			
Sample size	DE	DP	DM	DB
50	0.719	0.801	0.557	0.665
75	0.760	0.732	0.576	0.698
100	0.782	0.678	0.599	0.706
500	0.816	0.550	0.583	0.646

Average correct classification rate for detected DD genes. Averages are calculated over 20 replications. Standard errors were <0.025 (not shown)

*DB* both differential modality and different component means, *DD* differential distribution, *DE* differential expression, *DM* differential modality, *DP* differential proportion

Since the ability toclassify a DD gene correctly depends on the ability to detect the correct number of components (see classification algorithm in “Methods”), we also examine how the correct classification rate varies with component mean distance for the categories that contain bimodal genes (DP, DM, and DB). As shown in Table 4, the classification rates improve as *Δ*_*μ*_ increases. This pattern mirrors the trend in Table 1, and suggests that misclassification events occur largely due to incorrect estimation of the number of components. Performance generally increases with sample size, especially at lower values of *Δ*_*μ*_. In general, the ability of the algorithm to classify detected DD genes into their true category is robust when components are well separated and improves with increasing sample size.

**Table 4:** Average correct classification rates by component mean distance

Sample size	Gene category	Component mean distance *Δ*_*μ*_				
		2	3	4	5	6
50	DP	0.02	0.20	0.78	0.94	0.98
	DM	0.10	0.23	0.59	0.81	0.89
	DB	0.08	0.22	0.59	0.80	0.80
75	DP	0.02	0.18	0.77	0.94	0.97
	DM	0.08	0.27	0.69	0.86	0.90
	DB	0.09	0.29	0.71	0.83	0.84
100	DP	0.03	0.16	0.74	0.93	0.95
	DM	0.10	0.32	0.76	0.87	0.91
	DB	0.08	0.32	0.80	0.85	0.84
500	DP	0.01	0.15	0.72	0.91	0.93
	DM	0.12	0.33	0.72	0.85	0.89
	DB	0.03	0.43	0.85	0.85	0.85

Average correct classification rates stratified by *Δ*_*μ*_. Averages are calculated over 20 replications. Standard errors were <0.025 (not shown)

*DB* both differential modality and different component means, *DM* differential modality, *DP* differential proportion

### Case study: identifying DD genes between hESC types

The comprehensive characterization of transcriptional dynamics across hESC lines and derived cell types aims to provide insight into the gene regulatory processes governing pluripotency and differentiation [37–39]. Previous work utilizing microarrays and bulk RNA-seq largely focused on identifying genes with changes in average expression level across a population of cells. By examining transcriptional changes at the single-cell level, we can uncover global changes that are undetectable when averaging over the population. In addition, we gain the ability to assess the level of heterogeneity of key differentiation regulators, which may lead to the ability to assess variation in pluripotency [40] or the differentiation potential of individual cells.

The number of significant DD genes for each cell type comparison is shown in Table 5 for scDD, SCDE, and MAST. Note that the comparison of H1 and H9 detects the fewest number of DD genes for all three methods, a finding that is consistent with that both of these are undifferentiated hESC lines and it is expected that they are the most similar among the comparisons. In all four comparisons, the number of genes identified by our method is greater than that for SCDE and similar to that for MAST.

**Table 5:** Number of DD genes identified in the hESC case study data for scDD, SCDE, and MAST

	scDD							
Comparison	DE	DP	DM	DB	DZ	Total	SCDE	MAST
H1 vs NPC	1686	270	902	440	1603	5555	2921	5887
H1 vs DEC	913	254	890	516	911	5295	1616	3724
NPC vs DEC	1242	327	910	389	2021	5982	2147	5624
H1 vs H9	260	55	85	37	145	739	111	1119

Note that the total for scDD includes genes detected as DD but not categorized

*DB* both differential modality and different component means, *DD* differential distribution, *DE* differential expression, *DEC* definitive endoderm cell, *DM* differential modality, *DP* differential proportion, *DZ* differential zeroes, *hESC* human embryonic stem cell, *NPC* neuronal progenitor cell

**Fig. 5:**
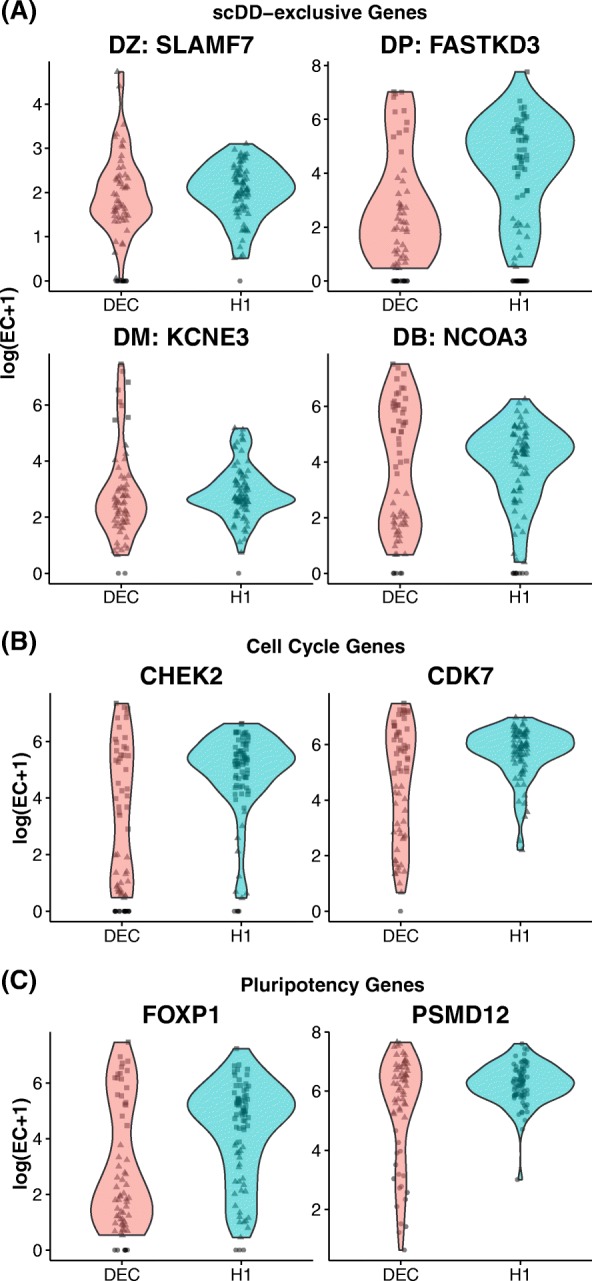
Violin plots (smoothed non-parametric kernel density estimates) for Differentially Distributed genes identified between H1 and DEC. Individual observations are displayed with jitter. Within a condition, points with the same shape are predicted to belong to the same component. **a** scDD-exclusive genes: representative genes from each category (DZ, DP, DM, and DB) that are not detected by MAST or SCDE. Selected genes are top-ranked by permutation *p* value in each category (DP, DM, and DB) or had a significant *χ* ^2^ test for a difference in the proportion of zeroes (DZ). **b** Cell-cycle genes: DD genes involved in cell-cycle regulation (not detected by MAST or SCDE). **c** Pluripotency genes: DD genes involved in pluripotency regulation (not identified by MAST or SCDE). *DB* both differential modality and different component means, *DD* differential distribution, *DEC* definitive endoderm cell, *DM* differential modality, *DP* differential proportion, *DZ* differential zeroes

Figure 5 a displays top-ranked genes for each category that are not identified by MAST or SCDE for the H1 versus DEC comparison. Among the genes identified exclusively by scDD for the H1 versus DEC comparison are *CHEK2*, a cell-cycle checkpoint kinase [41], and *CDK7*, a cyclin-dependent kinase that plays a key role in cell-cycle regulation through the activation of other cyclin-dependent kinases [42]. It has been shown that embryonic stem cells express cyclin genes constitutively, whereas in differentiated cells, cyclin levels are oscillatory [43]. This finding is consistent with the differential modality of the *CDK7* gene shown in Fig. 5 b. Similarly, scDD identifies several genes involved in the regulation of pluripotency that are not identified by the other two methods (Fig. 5 c). For example, *FOXP1* exhibits alternative splicing activity in hESCs, stimulating expression of several key regulators of pluripotency [44]. The *PSMD12* gene encodes a subunit of the proteasome complex that is vital to the maintenance of pluripotency and has shown decreased expression in differentiating hESCs [45]. Both of these genes are also differentially distributed between H1 and the other differentiated cell type, NPC.

In general, the vast majority of the genes found exclusively by scDD are categorized as something other than DE (ranging from 98.3 to 100 *%* in the three case studies, see Additional file 1: Table S6), which suggests that they are predominantly characterized by differences that are more complex than the traditional DE pattern. The genes identified by MAST but not scDD are overwhelmingly characterized as those with a weak signal in both the nonzero and zero components (see Additional file 1: Figure S9), which can be difficult to interpret (see Additional file 1: Section 3 for more details).

### Additional case studies

We also applied scDD and MAST to two additional case studies (the numbers of significant DD genes for each comparison are displayed in Table 6). SCDE was not used to analyze these datasets since it is intended for use on raw count data and the processed data made available by the authors of [31, 32] were already normalized by FPKM and TPM, respectively. Like the results of the hESC case study, MAST and scDD identify similar numbers of significant genes. The genes that scDD finds exclusively are predominantly characterized by something other than a mean shift, a result which is also consistent with the hESC case study (see Additional file 1: Table S7).

**Table 6:** Number of DD genes identified in the myoblast and mESC case studies for scDD and MAST

	scDD						
Comparison	DE	DP	DM	DB	DZ	Total	MAST
Myoblast: T0 vs T72	312	44	200	36	1311	2134	2904
mESC: Serum vs 2i	5233	76	1259	1128	670	9130	9706

Note that the total for scDD includes genes detected as DD but not categorized

*DB* both differential modality and different component means, *DD* differential distribution, *DE* differential expression, *DM* differential modality, *DP* differential proportion, *DZ* differential zeroes, *mESC* mouse embryonic stem cell

### Advantages and limitations of the approach

We stress that our approach is inherently different from a method that detects traditional DE, such as [17] and [18], which aim to detect a shift in the mean of the expressed values. In addition to identifying genes that have DDs across conditions, our modeling framework allows us to identify subpopulations within each condition that have differing levels of expression of a given gene (i.e., which cells belong to which component). For such genes, the partition estimates automatically provide an estimate of the proportion of cells in each condition that belong to each subpopulation. We also do not require specification of the total number of components, which can vary for each gene.

When applied to cells at different differentiation stages, this information may provide insight into which genes are responsible for driving phenotypic changes. The gene in Fig. 3 b, for example, shows a DP of cells across conditions, which is important to recognize since DP suggests a change in cell-specific responses to signaling [7, 29]. This is in contrast to the DM gene in Fig. 3 c, which indicates the presence of a distinct cell type in one condition, but not the other. Recent methods for scRNA-seq [17, 18, 27, 28, 46] may be able to identify genes such as those shown in Fig. 3 b–d as differing between conditions. However, our simulations suggest that they would be relatively underpowered to do so, and they would be unable to characterize the change as DP, DM, or DB.

We also show through simulation that our approach can accommodate large sample sizes of several hundreds of cells per condition. Note, however, that the real strength in the modeling framework lies in the ability to characterize patterns of DDs. In the presence of extreme sparsity, this will be a challenge, since the number of nonzero observations in a given gene will be small. If the sample size of nonzero measurements is too small, it will be difficult to infer the presence of multiple underlying cell states. In practice, for larger and more sparse datasets, it is recommended to verify that the number of cells expressing a given gene is in the range of the sample sizes considered in this study to utilize fully the available features of scDD.

The approach is limited in that adjustments for covariates are not directly incorporated into the model. In general, when the relationship between a potential confounding variable and the quantification of expression is well known (e.g., increased sequencing depth is generally associated with increased expression measurements), this should be accounted for in a normalization procedure. For other covariates that are not as well characterized (e.g., the cellular detection rate and batch effects), residuals can be used in the permutation procedure, though a more unified approach would be desirable. We also note that more complex confounding variables may be present in scRNA-seq experiments that are nonlinear in nature (e.g., covariate-specific effects on variance). We show in Additional file 1: Section 2.3 that when these effects are extreme, care must be taken in interpreting DD genes that are uncategorized.

Additionally, the approach is limited in that only pairwise comparisons across biological conditions are feasible. While an extended Bayes factor score to test for the dependence of a condition on a partition estimation for more than two conditions would be straightforward, the classification into meaningful patterns would be less so, and work is underway in that direction. Finally, we note that while the genes identified by scDD may prove useful in downstream analysis, interpretability is limited as partitions are estimated independently for each gene and consequently do not provide a unified clustering of cells based on global gene expression changes. Extensions in this direction are also underway.

## Conclusions

To our knowledge, we have presented the first statistical method to detect differences in scRNA-seq experiments that explicitly accounts for potential multi-modality of the distribution of expressed cells in each condition. Such multi-modal expression patterns are pervasive in scRNA-seq data and are of great interest, since they represent biological heterogeneity within otherwise homogeneous cell populations; differences across conditions imply differential regulation or response in the two groups. We have introduced a set of five interesting patterns to summarize the key features that can differ between two conditions. Using simulation studies, we have shown that our method has comparable performance to existing methods when differences (mean shifts) exist between unimodal distributions across conditions, and it outperforms existing approaches when there are more complex differences.

## Methods

### Software implementations and applications

All analyses were carried out using R version 3.1.1 [47]. The method MAST [18] was implemented using the MAST R package version 0.931, obtained from GitHub at https://github.com/RGLab/MAST. The adjustment for cellular detection rate as recommended in [18] was included in the case study, but not in the simulation study (only the normal component of the test was considered here since no difference in dropout rate was simulated). The method SCDE [17] was implemented using the scde R package version 1.0, obtained from http://pklab.med.harvard.edu/scde/index.html. No adjustment for cellular detection rate was carried out since SCDE cannot accommodate covariates. Since SCDE requires raw integer counts as input, and expected counts are non-integer valued, the ceiling function was applied to the unnormalized counts. For each approach, the target FDR was controlled at 5 %. Specifically, both MAST and SCDE provide gene-specific *p* values and use the method of [35] to control FDR. We followed the same procedure here.

Our method is implemented using version 1.1.0 of the scDD R package, available at https://github.com/kdkorthauer/scDD. The analysis involves a computationally intensive permutation step, which is executed in parallel on multiple cores if available. On a Linux machine using 12 cores and up to 16 gigabytes of memory, this step took approximately 60 minutes for 1000 permutations of 1000 genes in the simulation of 50 samples per condition. Computation time scales approximately linearly with sample size, and this same task takes approximately 90 minutes for 100 samples per condition, and 300 minutes for a sample size of 500 per condition. The computation time to analyze the simulated datasets for SCDE (MAST) ranged from approximately 3 to 30 (0.5 to 5) minutes across the different sample sizes.

### hESC culture and differentiation

All cell culture and scRNA-seq experiments were conducted as described previously [30, 48]. Briefly, undifferentiated H1 and H9 hESCs were routinely maintained at the undifferentiated state in E8 medium on Matrigel (BD Bioscience) coated tissue culture plates with daily medium feeding [49]. HESCs were passaged every 3 to 4 days with 0.5 mM ethylenediaminetetraacetic acid (EDTA) in phosphate-buffered saline (PBS) at 1:10 to 1:15 ratio for maintenance. H1 were differentiated according to previously established protocols [50, 51]. All the cell cultures performed in our laboratory have been routinely tested as negative for mycoplasma contamination.

For DECs, H1 cells were individualized with Accutase (Life Technologies), seeded in E8 with BMP4 (5 ng/ml), Activin A (25 ng/ml) and CHIR99021 (1 *μ*M) for the first 2 days, then withdraw CHIR99021 for the remaining period of differentiation. DECs were harvested at the end of day 5, and sorted for the CXCR4-positive population for scRNA-seq experiments. For NPCs, the undifferentiated H1-SOX2-mCherry reporter line was treated with 0.5 mM EDTA in PBS for 3 to 5 min and seeded in E6 (E8 minus FGF2, minus TGF *β*1), with 2.5 *μ*g/ml insulin, SB431542 (10 *μ*M) and 100 ng/ml Noggin. NPCs were harvested and enriched at the end of day 7, after sorting for the Cherry-positive population for scRNA-seq experiments. All differentiation media were changed daily.

### Read mapping, quality control, and normalization

For each of the cell types studied, expected counts were obtained from RSEM [52]. In each condition there are a maximum of 96 cells, but all have fewer than 96 cells due to removal by quality control standards. Some cells were removed due to cell death or doublet cell capture, indicated by a post cell capture image analysis as well as a very low percentage of mapped reads. For more details on read mapping and quality control, see [30, 48]. DESeq normalization [53] was carried out using the MedianNorm function in the EBSeq R package [54] to obtain library sizes. The library sizes were applied to scale the count data. Further, genes with a very low detection rate (detected in fewer than 25 *%* of cells in either condition) are not considered.

### Publicly available scRNA-seq datasets

Processed FPKM-normalized data from human myoblast cells [31] were obtained from GEO [55] using accession number GSE52529. In this study, we examined the set of cells cultured on standard growth medium (samples labeled with T0) as well as those treated with differentiation-inducing medium for 72 h (samples labeled with T72). Processed TPM-normalized data from mESCs [32] were also obtained from GEO under accession number GSE60749. In this study, we examined the samples labeled as mESC (cultured in standard medium), along with the samples labeled as TwoiLIF (cultured in 2i + LIF differentiation-inhibitory medium).

### Publicly available bulk RNA-seq datasets

The modality of the gene expression distributions in bulk RNA-seq was explored using large, publicly available datasets and the results are displayed in Fig. 2. In this figure, the red bars depict the bulk RNA-seq results, and datasets are labeled according to their source and sample size. Datasets GE.50, GE.75, and GE.100 are constructed by randomly sampling 50, 75, and 100 samples from GEUVADIS [56] to obtain sample sizes comparable to the single-cell sets under study (obtained from the GEUVADIS consortium data browser at www.ebi.ac.uk/arrayexpress/files/E-GEUV-1/analysis_results/GD660.GeneQuantCount.txt.gz). Dataset LC consists of 77 normal lung tissue samples from the TCGA lung adenocarcinoma study [57] (obtained from GEO [55] using accession number GSE40419). All datasets were normalized using DESeq normalization [53] except for LC, for which the authors supplied values already normalized by RPKM.

### Mixture model formulation

#### Dirichlet process mixture of normals

Let ${Y^{c}_{g}}=(y^{c}_{g1},\ldots,y^{c}_{g{J_{c}}})$ be the log-transformed nonzero expression measurements of gene *g* for a collection of *J*_*c*_ cells in condition *c* out of 2 total conditions. For simplicity of presentation, we drop the dependency on *g* for now, and let the total number of cells with nonzero measurements be *J*. We assume that under the null hypothesis of equivalent distributions (i.e., no dependency on condition), *Y*={*Y* ^*c*^}_*c*=1,2_ can be modeled by a conjugate DPM of normals given by

1 $$ \begin{aligned} {y^{c}_{j}} &\sim N(\mu_{j}, \tau_{j}) \\ \mu_{j}, \tau_{j} &\sim G \\ G &\sim \operatorname{DP}(\alpha, G_{0}) \\ G_{0} & = \operatorname{NG} (m_{0}, s_{0}, a_{0}/2, 2/b_{0}) \\ \end{aligned}  $$

where DP is the Dirichlet process with base distribution *G*_0_ and precision parameter *α*, *N*(*μ*_*j*_,*τ*_*j*_) is the normal distribution parameterized with mean *μ*_*j*_ and precision *τ*_*j*_ (i.e., with variance $\tau _{j}^{-2}$), and NG(*m*_0_,*s*_0_,*a*_0_/2,2/*b*_0_) is the normal-gamma distribution with mean *m*_0_, precision *s*_0_ *τ*_*j*_, shape *a*_0_/2, and scale 2/*b*_0_. Let *K* denote the number of components [unique values among $(\mu, \tau) = \{\mu _{j}, \tau _{j}\}_{j=1}^{J}$]. Note that two observations indexed by *j* and *j* ^′^ belong to the same component if and only if $(\mu _{j}, \tau _{j})=(\mu _{j^{\prime }}, \phantom {\dot {i}\!}\tau _{j^{\prime }})$.

#### Product partition models

The posterior distribution of (*μ*,*τ*) is intractable even for moderate sample sizes. This is because the number of possible partitions (clusterings) of the data grows extremely rapidly as the sample size increases (according to the Bell number). However, if we let *Z*=(*z*_1_,…,*z*_*J*_) be the vector of component memberships of gene *g* for all samples, where the number of unique *Z* values is *K*, the probability density of *Y* conditional on *Z* can be viewed as a PPM [58, 59]. Thus, it can be written as a product over all component-specific densities:

2 $$ f(Y|Z) = \prod_{k=1}^{K} f(y^{(k)}) \\  $$

where *y* ^(*k*)^ is the vector of observations belonging to component *k* and *f*(*y* ^(*k*)^) is the component-specific distribution after integrating over all other parameters. In the conjugate normal-gamma setting, this has a closed form given by

3 $$ f(y^{(k)}) \propto \frac{\Gamma(a_{k}/2) }{(b_{k}/2)^{a_{k}/2}} s_{k}^{-1/2}.  $$

The posterior for the parameters (*μ*_*k*_,*τ*_*k*_) conditional on the partition is

4 $$ (\mu_{k}, \tau_{k}) | Y, Z \sim \operatorname{NG} (m_{k}, s_{k}, a_{k}/2, 2/b_{k}).  $$

The posterior parameters (*m*_*k*_, *s*_*k*_, *a*_*k*_, *b*_*k*_) also have a closed form due to the conjugacy of the model given by Eq. 1. These parameters are given by

5 $$ \begin{aligned} s_{k} &= s_{0} + n^{(k)} \\ m_{k} &= \frac{s_{0} m_{0} + \sum y^{(k)}}{s_{k}} \\ a_{k} &= a_{0} + n^{(k)} \\ b_{k} &= b_{0} + \sum (y^{(k)})^{2} + s_{0}{m_{0}^{2}} - s_{k}{m_{k}^{2}} \\ \end{aligned}  $$

where *n* ^(*k*)^ is the number of observations in component *k*. It follows that the marginal posterior distribution of *μ*_*k*_ conditional on the partition is

6 $$ \mu_{k} | Y, Z \sim t_{a_{k}} \left(m_{k}, \frac{b_{k}}{a_{k} s_{k}}\right)  $$

where *t*_*a*_(*b*,*c*) denotes the generalized Student’s *t* distribution with *a* degrees of freedom, noncentrality parameter *b*, and scale parameter *c*. The product partition DPM model can be simplified as follows:

7 $$ \begin{aligned} y_{j} \, | z_{j}=k, \mu_{k}, \tau_{k} &\sim N(\mu_{k}, \tau_{k}) \\ \mu_{k}, \tau_{k} &\sim \operatorname{NG} (m_{0}, s_{0}, a_{0}/2, 2/b_{0}) \\ z &\sim \frac{\alpha^{K} \Gamma(\alpha)}{\Gamma(\alpha+J)} \prod_{k=1}^{K} \Gamma(n^{(k)}). \end{aligned}  $$

Then we can obtain the joint predictive distribution of the data *Y* and partition *Z* by incorporating Eq. 7:

8 $$ \begin{aligned} f(Y, Z) &= f(Z) \prod_{k=1}^{K} f(y^{(k)}) \\ & \propto \alpha^{K} \prod_{k=1}^{K} \frac{\Gamma(n^{(k)}) \Gamma(a_{k}/2) }{ (b_{k}/2)^{a_{k}/2}} s_{k}^{-1/2}. \end{aligned}  $$

#### Model-fitting

The fitting of the model given in Eq. 7 involves obtaining an estimate $\hat {Z}$ of the partition. The goal is to find the partition that yields the highest posterior mass in Eq. 8, referred to as the maximum a posteriori (MAP) partition estimate. Under this modeling framework, the solution for the MAP estimate is not deterministic and several computational procedures have been developed utilizing Polya urn Gibbs sampling [60–62], agglomerative greedy search algorithms [63, 64], or an iterative stochastic search [65].

These procedures generally involve evaluation of the posterior at many different candidate partitions, and as such tend to be computationally intensive. To avoid this challenge, we recognize the relation to the corresponding estimation problem in the finite mixture model framework, where the partition estimate can be obtained by optimizing the Bayesian information criterion (BIC) of the marginal density *f*(*Y*|*Z*) [66]. In fact, for certain settings of the prior distribution over partitions, the MAP estimate is identical to the estimate obtained by optimizing the BIC [59]. In practice, even when these settings are not invoked, the performance of partition estimates obtained via BIC optimization show comparable performance (see Additional file 1: Section 1). We obtain the partition estimate $\hat {Z}$ that optimizes the BIC using the Mclust R package [66] and satisfies the criteria for multi-modality described in the next section.

The hyperparameters for the component-specific mean and precision parameters were chosen so as to encode a heavy-tailed distribution over the parameters. Specifically, the parameters were set to *μ*_0_=0, ${\tau _{0}^{2}}=0.01$, *a*_0_=0.01, and *b*_0_=0.01. The Dirichlet concentration parameter was set to *α*=0.01, and choosing this is shown in Additional file 1: Section 1 to be robust to many different settings in a sensitivity analysis.

#### Partition estimation

The partition estimate $\hat {Z}$ is obtained that optimizes BIC using Mclust [66], in addition to the following filtering criteria. Note that the only constraint imposed on the number of components *K* in the modeling framework is that *K*≤*J*. However, under the sample sizes in this study, we consider only *K*≤5. The first filtering criterion is based on the notion that a two-component mixture model is not necessarily bimodal [67], and relaxes the requirement that the MAP estimate corresponds to the model with the lowest BIC. Specifically, for each candidate model fitted by BIC with *K* components, a split step (if *K*=1, obtain a new partition estimate $\hat {Z}$ with *K*=2 unique elements) or a merge step (if *K*≥2, obtain a new partition estimate $\hat {Z}$ restricted to *K*−1 unique elements) is carried out to generate a new candidate partition. The candidate partition with the larger value of *K* becomes the partition estimate only if the component separation suggests multi-modality. Component separation between any pair of components is assessed with the bimodality index (BI) [68]:

$$ \text{BI} = 2 \times \sqrt{\frac{n_{1} n_{2}}{(n_{1}+n_{2})^{2}}} \left(\frac{| \mu_{1} - \mu_{2} | }{\sigma} \right) $$

where the component means *μ*_1_ and *μ*_2_ are estimated via maximum likelihood, the common within-component standard deviation *σ* is conservatively estimated with the maximum within-component standard deviation among all components, and *n*_1_ and *n*_2_ are the number of cells belonging to each component. BI thresholds for the split and merge step were determined empirically and vary by sample size, as multiple modes are more easily detected as sample size increases [68] (for more details see Additional file 1: Section 4).

The second filtering criterion is designed to reduce the impact of outlier cells. Specifically, components with fewer than three cells are not considered, and the merge step is also carried out if one of the components present has an extremely large variance compared to the others (more than 20 times larger than any other component). Likewise, the split step is not carried out if one of the proposed components has a variance more than 10 times larger than any other component.

### Simulation details

#### Component means and variances

Each gene was simulated based on the characteristics of a randomly sampled unimodal gene with at least 25 *%* nonzero measurements in the H1 dataset. For unimodal genes, the mean and variance were chosen to match the observed mean and variance; for bimodal genes, the component means and variances were selected to be near the observed mean and variance. The proportion of zeroes is chosen to match that observed in the randomly sampled gene, and is not varied by condition. Details are provided in the following sections.

Distances between (log-scale) component means *Δ*_*μ*_ *σ* in the multi-modal genes were chosen such that components were separated by a minimum of two and a maximum of six standard deviations, where the standard deviation *σ* is assumed constant (on the log-scale) across components. The specific values of *σ* used for the simulated genes are empirical estimates of the standard deviations of the unimodal case study genes (assuming a lognormal distribution on the raw scale). In this setting, the component distance can also be thought of as a fold-change within condition (across components), where the ratio of the component means (untransformed-scale) is equal to $\mathrm {e}^{\Delta _{\mu }\hat {\sigma }}$. The ratio of the component standard deviations (raw scale) is also equal to this same fold-change (see Additional file 1: Section 2.1 for more details). The component mean distance values were chosen to represent a range of settings for which the difficulty of detecting multi-modality is widely varied, as well as to reflect the range of observed component mean distances detected empirically in the case studies.

#### Unimodal genes

The parameters of the negative binomial distribution for unimodal genes were estimated from the randomly sampled observed genes using the method of moments. These empirical parameters were used as is to simulate both conditions of EE genes, and condition 1 of DE and DB. Condition 1 of DM was simulated by decreasing the mean by half the value of *Δ*_*μ*_. The second condition for DE genes was simulated based on condition 1 parameters using randomly sampled fold-changes that were between two and three standard deviations of the observed fold-changes between H1 and DEC.

#### Bimodal genes

The parameters for the mixture of negative binomial distributions in bimodal genes were also generated using empirically estimated means and variances. The first (lower) component mean was decreased by half the value of *Δ*_*μ*_ and the second (higher) component mean was increased by half the value of *Δ*_*μ*_.

### DD classification algorithm

Genes detected as significantly DD from the permutation test of the Bayes factor score are categorized into patterns of interest. The genes that are not classified as DE, DP, DM, or DB are considered to be no calls, abbreviated NC. These represent patterns that are not of primary interest, such as those that differ only in variance (but not in the number of components or their means). This type of difference may result from cell-specific differences in technical variation [17], which can only be decomposed from biological variation in experimental protocols that allow for independent estimation of technical effects using spike-in controls, for example [69].

An additional step to improve the power to detect genes in the DP category was also implemented. This step was motivated by the observation that the Bayes factor score tends to be small when the clustering process within each condition is consistent with that overall, as in the case of DP. Thus, for genes that were not significantly DD by permutation but had the same number of components within condition as overall, Fisher’s exact test was used to test for independence with biological condition. If the *p* value for that test is less than 0.05, then the gene was added to the DP category (this did not result in the addition of any false positives in the simulation study). In addition, since the Bayes factor score depends on the estimated partition, we increase the robustness of the approach to detect DD genes under possible misspecification of the partition by also assessing evidence of DD in the form of an overall mean shift for genes not significant by the permutation test (using a *t*-statistic with FDR controlled by [35]). This resulted in the detection of between 121 and 689 additional genes in the hESC comparisons and did not add any false positives in 94 *%* of simulation replications (with only a single false positive gene in the other 6 *%* of replications).

Here we present pseudocode for the classification of DD genes into the categories DE, DP, DM, or DB. For every pair of components, we obtain a sample of 10,000 observations from the posterior distribution of the difference in means. The components are considered to overlap if the 100 *%* credible interval contains 0.

### DD classification algorithm



## Additional file

Additional file 1 Supplement. Sensitivity analyses of MAP estimation method, further methodological details, and additional results. (PDF 553 kb)

## Abbreviations

BIC: Bayesian information criterion
DD: differential distribution
DE: Differential expression
DEC: Definitive endoderm cell
DP: Differential proportion
DM: Differential modality
DB: Both differential modality and different component means
DPM: Dirichlet process mixture
DZ: Differential zeroes
ED: Equivalent distribution
EDTA: Ethylenediaminetetraacetic acid
EE: Equivalent expression
EP: Equivalent proportion
FDR: False discovery rate
hESC: Human embryonic stem cell
mESC: Mouse embryonic stem cell
MAP: Maximum a posteriori
NC: no call
NPC: Neuronal progenitor cell
PBS: Phosphate-buffered saline
PPM: Product partition model
scDD: Single-cell differential distributions
scRNA-seq: Single-cell RNA sequencing
