# Supplementary material for: A statistical approach for identifying differential distributions in single-cell RNA-seq experiments
Source: Genome Biol. 2016 Oct 25;17:222. doi: 10.1186/s13059-016-1077-y (PMC5080738; doi:10.1186/s13059-016-1077-y)
Supplement: Additional file 1 — Supplement. Sensitivity analyses of MAP estimation method, further methodological details, and additional results. (PDF 553 kb) [file 13059_2016_1077_MOESM1_ESM.pdf]

Supplementary Materials for "A statistical approach for identifying differential distributions in single-cell RNA-seq experiments"

1 MAP partition estimation sensitivity analysis

Here we present a sensitivity analysis to evaluate several computational approaches to obtain an estimate of the maximum a priori (MAP) partition in the Dirichlet process mixture model framework. Specifically, we use simulated data from mixtures of normals to compare an agglomerative greedy search algorithm [Ward Jr, 1963, Wang and Dunson, 2011], a Polya urn Gibbs sampling scheme [MacEachern, 1994, Bush and MacEachern, 1996, MacEachern and Müller, 1998], an iterative stochastic search [Shotwell, 2013], as well as a simple BIC optimization [Fraley et al., 2012]. The latter is implemented in the R package `Mclust`. The first three methods are implemented in the R package `profdpm` by Shotwell [2013].

In this sensitivity analysis we evaluate the ability of each procedure to detect the existence of multiple components when they truly exist as well as its false positive rate in detecting multiple components when there is only one. Since the expected number of components in the Dirichlet process mixture model depends on the value of α (the Dirichlet concentration parameter) as well as the sample size J [Antoniak, 1974], we vary both of these parameters. For each procedure, we evaluate each choice of $J \in (30, 50, 100, 200)$ and $\alpha \in (0.001, 0.01, 0.05, 0.10, 1, 5)$ under four scenarios:

1. **Null Scenario:** samples are drawn from a standard normal distribution
2. **Two close components:** samples are drawn from a mixture of two normals (equal weight), one standard normal and one with mean equal to two and variance equal to one
3. **Two moderately-separated components:** samples are drawn from a mixture of two normals (equal weight), one standard normal and one with mean equal to four and variance equal to one

4. **Two well-separated components:** samples are drawn from a mixture of two normals (equal weight), one standard normal and one with mean equal to six and variance equal to one

Each combination of α, J and scenario was evaluated for 100 replications. For the agglomerative, gibbs, and stochastic methods, the hyperparameters for the component-specific mean and precision parameters were chosen so as to encode a heavy-tailed distribution over the parameters ($\mu_0 = 0$, $\tau_0^2 = 0.01$, and $b_0 = 0.01$). Note that the partition estimates by Mclust do not depend on an α value or hyperparameters; instead they represent a particular setting of the prior distribution over partitions [Shotwell and Slate, 2011]. The gibbs and stochastic methods were implemented with default input settings for the number of iterations. Mclust was constrained to a maximum of 5 components.

Each scenario is assessed by its misclassification rate (how often the MAP estimate identifies something other than the true number of components). These results are shown in Figures S1 - S4. Results are stratified by values of J and, where applicable, by α. Note that the agglomerative method appears to be the most sensitive to sample size, whereas the gibbs procedure appears to be the most sensitive to the concentration parameter α. In addition, the Mclust results do not depend on α and are only strongly dependent on sample size when components are moderately spaced, but are generally as good as or better than the best α setting for each of the other procedures.

In addition, each scenario is assessed by the concordance of the MAP clustering estimates with the true partition, measured using the Rand index (proportion of concordant observation pairs). A higher Rand index represents better partition estimates. Figures S5-S8 display boxplots for the rand index for sample size 100 (results are similar for other values of J) across α values where applicable. Again, we note that the Mclust partition concordance results are generally as good as or better than the best α setting for each of the other procedures.

When components are tightly spaced, as in Scenario 2, the misclassification rate is high and concordance is low for all four procedures. These results suggest that the Dirichlet process mixture model is not reliable for detecting the existence of more than one component when the components are very close together. We also conclude that the Mclust procedure performs as well as the other methods, and has the added advantage that prior parameters do not need to be specified. Computation time is also greatly reduced compared to the gibbs and stochastic procedures, which require evaluating the posterior over many iterations. Note that although the partition estimation by Mclust does not require specification of α, the evaluation of the differentially distributed score does depend on this value, as it represents our prior belief in the number of components present. Here we choose $\alpha = 0.01$, as this value appears the most robust in balancing misclassification rates among the four scenarios for the agglomerative, gibbs, and stochastic procedures.

2 Additional details of negative binomial simulations

2.1 Interpretation of component mean distance

The scDD framework assumes that log-transformed gene specific expression distributions can be modeled by a mixture of normals. One of the aims of the simulation study in the main text is to evaluate the sensitivity of results to the degree of separation of mixture components, which is varied by specifying Δ_μ. Note that although the scDD modeling framework assumes a continuous mixture of normal distributions, we evaluate performance based on counts simulated from mixtures of negative binomials. The interpretation of Δ_μ depends on the scale of the data (raw counts versus log-transformed values). On the log-scale, this parameter represents the number of standard deviations that separate the means of two components. On the raw count scale, Δ_μ can instead be thought of in terms of a fold change. This can be seen by examining Δ_μ in terms of parameters of the lognormal distribution.

For a two-component mixture of normals with component means μ_1 and μ_2 (where $\mu_2 > \mu_1$) and common standard deviation σ, we let $\Delta_\mu \sigma = \mu_2 - \mu_1$. On the untransformed scale, these two components follow a lognormal distribution with means m_1 and m_2 (where $m_2 > m_1$), and standard deviations s_1 and s_2, respectively. Note that for the lognormal distribution the relationship between the log-scale parameters (μ, σ) and the untransformed parameters $(m,\, s)$ is

$$m = e^{\mu+\sigma^2/2}, \text{ and}$$
$$s = \sqrt{(e^{\sigma^2}-1)e^{2\mu+\sigma^2}}.$$

Then, since $\mu_2 = \mu_1 + \Delta_\mu \sigma$, the log fold change on the untransformed scale is

$$\begin{aligned} log\left(\frac{m_2}{m_1}\right) &= \mu_2 + \sigma^2/2 - \mu_1 - \sigma^2/2 \\ &= \mu_1 + \Delta_\mu \sigma - \mu_1 \\ &= \Delta_\mu \sigma \end{aligned}$$

Additionally, the log ratio of the untransformed standard deviations is

$$
\begin{aligned}
log\left(\frac{s_2}{s_1}\right) &= log\left(\sqrt{\frac{(e^{\sigma^2}-1)e^{2\mu_2+\sigma^2}}{(e^{\sigma^2}-1)e^{2\mu_1+\sigma^2}}}\right) \\
&= log\left(\sqrt{\frac{e^{2(\mu_1+\Delta_\mu\sigma)}}{e^{2\mu_1}}}\right) \\
&= log\left(\sqrt{e^{2\Delta_\mu\sigma}}\right) \\
&= \Delta_\mu\sigma
\end{aligned}
$$

The fold change depends on both the component mean distance parameter Δ_μ and the component standard deviation estimate (on the log-scale) $\hat{\sigma}$. In practice, for each gene from the case study that is used for parameter estimation in the simulations, $\hat{\sigma}$ is estimated assuming a log-normal distribution:

$$
\hat{\sigma} = \sqrt{log\left(1 + \frac{\hat{s}^2}{\hat{m}^2}\right)}
$$

where $\hat{m}$ and $\hat{s}$ are the estimated sample mean and sample standard deviation of the untransformed nonzero measurements. In practice, the values obtained from the case study datasets used in the simulation ranged from 0.21 to 1.80 with a median value of 0.86. Since $\hat{\sigma}$ is typically less than 1, the log fold change is typically less than Δ_μ.

The specific values of component mean distance were chosen to represent a range of settings for which the difficulty of detecting multi-modality is widely varied in simulation, as well as to reflect the range of observed component mean distances detected empirically in the case studies. Specifically, simulation studies in the previous section demonstrate that it is very difficult to detect multiple components when they are separated by only 2 standard deviations on the log scale. However, this task becomes easier as the separation increases up to 6 standard deviations on the log scale, and results do not differ substantially outside of this range. In addition, these settings cover the range of component mean distances observed in case studies. Specifically, the median component mean distance for the 1883 genes detected as having two components in the H1 dataset was 3.93, with the 1st and 99th percentile being 2.49 and 5.93, respectively. The standard deviation here is computed as the square root of the weighted average of estimated component variances.

2.2 Additional Simulation Results

Here we examine the estimation of the number of components within condition in the simulation study from the main text. The results, separated by gene category, are shown in Table S1. The settings with one component in each condition (EE and DE) have the highest correct detection rates, whereas lower rates are observed in the more difficult cases with more than one component in both conditions (EP and DP).

		True Gene Category					
Sample Size	Condition	EE	EP	DE	DP	DM	DB
50	1	0.907	0.519	0.910	0.509	0.912	0.907
	2	0.908	0.520	0.900	0.511	0.521	0.547
75	1	0.906	0.578	0.909	0.571	0.916	0.904
	2	0.909	0.580	0.907	0.570	0.578	0.606
100	1	0.905	0.618	0.905	0.602	0.907	0.901
	2	0.905	0.618	0.902	0.601	0.616	0.640
500	1	0.882	0.690	0.885	0.658	0.877	0.884
	2	0.883	0.691	0.901	0.661	0.688	0.683

Table S1: Average proportion of simulated genes in each category where the correct number of components was identified. Averages are calculated over 20 replications. Standard errors were < 0.02 (not shown).

Next, we examine the ability of scDD as a whole to detect *and* classify each DD gene into its corresponding category. Here, power is defined as the proportion of genes detected and classified correctly. We also define two different types of FDR to differentiate between a false discovery at the detection stage and a false discovery at the classification stage. Briefly, for a given category we let

$$FDR_{\text{detect}} = \frac{\text{number of null genes assigned to that category}}{\text{total number of genes assigned to that category}}$$

$$FDR_{\text{classify}} = \frac{\text{number of non-null genes assigned that belong to a different category}}{\text{total number of genes assigned to that category}}$$

Results are shown in Table S2. The FDR_{classify} is inflated in the DE, DB, and DM cases, meaning that a substantial proportion of the DD genes classified as DE, DB, or DM belong to another category. As shown in the main text (Table 4), the majority of the misclassified genes tend to belong to low component mean distance settings. Examining these events in more detail, it is the case that the majority of them occur when the correct number of components is not identified. In general, the ability of the algorithm to detect

and classify DD genes into their true category is robust when components are well-separated and improves with increasing sample size.

Sample Size	Statistic	Gene Category DE	DP	DM	DB
50	Power	0.642	0.335	0.500	0.380
	FDR_{detect}	0.002	0.019	0.026	0.027
	FDR_{classify}	0.209	0.028	0.248	0.131
75	Power	0.723	0.432	0.553	0.466
	FDR_{detect}	0.001	0.024	0.026	0.033
	FDR_{classify}	0.248	0.010	0.215	0.166
100	Power	0.760	0.486	0.588	0.513
	FDR_{detect}	0.001	0.028	0.021	0.034
	FDR_{classify}	0.278	0.007	0.202	0.194
500	Power	0.815	0.541	0.583	0.584
	FDR_{detect}	0.001	0.052	0.002	0.023
	FDR_{classify}	0.394	0.005	0.112	0.310

Table S2: Average power to detect and classify simulated DD genes by category. Averages are calculated over 20 replications. Standard errors were < 0.025 (not shown).

2.3 Sensitivity analysis of confounded technical variance

The procedure to adjust for known confounders using a linear modeling framework as described in the main manuscript is aimed at adjusting for confounding effects that impact the magnitude of expression levels. However, it is possible that covariate-specific technical effects exist that affect the variance of expression levels rather than the mean. When such covariates are not balanced across biological conditions, the variance levels will be confounded.

To assess the impact of this type of nonlinear confounding effect, we carried out a sensitivity analysis to evaluate the effect on power and false discovery rate in the extreme case when there exists a covariate-specific difference in variance that is completely confounded with biological condition. Specifically, we repeated the simulation described in the main manuscript and let the log-scale variance in one condition be inflated by a common factor for all cells in one condition, while leaving the variance as is in the other condition. The log-scale means were also kept the same as in the original simulation study. We then evaluated overall power and false discovery rate for scDD as well as existing methods MAST and SCDE.

Results for the sensitivity study using variance inflation factors of 1.10, 1.25, and 1.5 are shown in Tables S3, S4, and S5, respectively. In the presence of confounded covariate-

Sample Size	scDD Including NC	Excluding NC	SCDE	MAST
50	0.687 (0.034)	0.561 (0.017)	0.489 (0.004)	0.546 (0.024)
75	0.789 (0.036)	0.666 (0.015)	0.576 (0.003)	0.638 (0.025)
100	0.844 (0.041)	0.730 (0.018)	0.627 (0.003)	0.686 (0.026)
500	0.972 (0.095)	0.856 (0.022)	0.906 (0.008)	0.792 (0.036)

Table S3: Average power (FDR) to detect simulated DD genes in the presence of a covariate-specific variance confounding (inflation factor = 1.10). Averages are calculated over 20 replications. Standard errors were < 0.025 (not shown).

Sample Size	scDD Including NC	Excluding NC	SCDE	MAST
50	0.680 (0.049)	0.555 (0.019)	0.485 (0.005)	0.541 (0.030)
75	0.778 (0.067)	0.661 (0.025)	0.570 (0.006)	0.631 (0.034)
100	0.836 (0.079)	0.721 (0.026)	0.627 (0.005)	0.681 (0.036)
500	0.969 (0.419)	0.850 (0.079)	0.894 (0.047)	0.796 (0.095)

Table S4: Average power (FDR) to detect simulated DD genes in the presence of a covariate-specific variance confounding (inflation factor = 1.25). Averages are calculated over 20 replications. Standard errors were < 0.025 (not shown).

Sample Size	scDD Including NC	Excluding NC	SCDE	MAST
50	0.677 (0.126)	0.551 (0.042)	0.495 (0.009)	0.537 (0.048)
75	0.776 (0.183)	0.652 (0.056)	0.575 (0.016)	0.624 (0.055)
100	0.834 (0.248)	0.712 (0.069)	0.631 (0.025)	0.672 (0.066)
500	0.963 (0.714)	0.832 (0.212)	0.875 (0.278)	0.810 (0.293)

Table S5: Average power (FDR) to detect simulated DD genes in the presence of a covariate-specific variance confounding (inflation factor = 1.50). Averages are calculated over 20 replications. Standard errors were < 0.025 (not shown).

specific variance effects, we see that the power to detect DD genes is still favorable compared to MAST and SCDE. For all three methods (scDD, MAST, and SCDE), the FDR is elevated at higher sample sizes. In addition, the degree of variance inflation also has an effect on the power for all three methods, with increasing inflation from 1.10 to 1.50 leading to slight decreases in power. We also note that there is an increase in the false discovery rate for scDD when the NC (or 'no call') genes are included. This FDR inflation arises because

the Bayes Factor detects a distributional difference when the variance is inflated in one condition compared to the other. As expected, however, the DD classification does not categorize these genes (NC, or 'no call' genes, are those which do not fall into the DE, DP, DM, or DB categories). This suggests that the distributional difference lies primarily in the variance (and not in the means or number of modes). As explained in the "DD classification algorithm" section, we do not aim to interpret these types of differences, since they can result from differences in covariate-specific technical variance. Identifying changes in biological variance (with no change in the number of components or their means) requires independent estimation of technical effects, which can be carried out with the method from Vallejos et al. [2016] for experimental protocols that accommodate spike-in control transcripts.

3 Additional results for hESC case studies

Here we examine the overlap of the differentially expressed genes identified by SCDE and MAST with the differentially distributed genes identified by scDD in the hESC case studies described in the main text. Table S6 shows the number of genes found exclusively by each method, and for scDD, the number of those that were classified into each pattern. Note that in two out of the four comparisons, MAST finds the most exclusive genes and in all comparisons finds SCDE the fewest. In addition, of the genes identified only by scDD, very few are categorized as the DE pattern. Since these scDD-exclusive genes predominantly exhibit more complex patterns than a mean shift, it is not surprising that they are not identified by SCDE or MAST. This result is also observed in the myoblast and mESC case studies (see Table S7).

	SCDE	MAST	scDD only					
Comparison	only	only	DE	DP	DM	DB	DZ	Total
H1 vs NPC	72	721	1	72	133	84	48	471
H1 vs DEC	132	505	36	151	378	303	10	2121
NPC vs DEC	71	535	0	123	215	122	99	959
H1 vs H9	8	473	1	31	14	20	6	104

Table S6: Number of genes found exclusively by SCDE, MAST, and scDD in hESC case study. Note that the Total for scDD includes genes detected as DD but not categorized

Like scDD, MAST identifies genes with a difference in average nonzero measurements as well as those with a difference in the proportion of zeroes. Specifically, the test statistic of MAST combines these two types of differential signal by summing them. This results in some genes with a weak signal in both the nonzero measurements (referred to as the "continuous" component) and in the proportion of zeroes (referred to as the "discrete"

Comparison	MAST only	scDD only DE	DP	DM	DB	DZ	Total
Myoblast T0 vs T72	891	2	12	53	6	6	121
mESC Serum vs 2i	774	13	4	47	10	36	198

Table S7: Number of genes found exclusively by MAST and scDD in the myoblast and mESC case studies. Note that the Total for scDD includes genes detected as DD but not categorized

component) to be identified as significantly differentially expressed by MAST. In fact, the MAST-exclusive genes can be predominantly characterized by a weak signal in both components. This is shown in Figure S9 for the Thomson hESC case study comparisons. Unlike MAST, scDD requires that a gene have a strong signal in at least one of the components by testing them independently. This is done in order to avoid detection of genes that have signal in opposite directions (e.g. higher mean expression in nonzero cells as well as a higher dropout rate). This type of pattern is contradictory to the observation that genes with higher expression typically have a higher capture rate, and thus is difficult to interpret.

4 Thresholding of bimodality index by sample size

The partition estimation step described in the Methods section of the main manuscript involves a model selection step by BIC combined with a filtering step that assesses the level of multi-modality for models with more than one component. Evidence of bimodality between any pair of components is measured with the Bimodality Index (BI) [Wang et al., 2009], which represents the number of standard deviations of separation between component means, multiplied by a balance factor (this factor decreases as imbalance in sample size of components increases). Note that we have multiplied the original definition of BI in Wang et al. [2009] by a factor of 2 so that when components are balanced the BI is simply the number of standard deviations of separation between the component means. As shown in Wang et al. [2009], as the sample size increases, the probability of identifying two components when the data-generating distribution is unimodal increases for a fixed threshold of BI. Thus to keep the false positive rate for identifying multi-modal distributions constant across a range of sample sizes, we use BI thresholds that vary with sample size.

The BI threshold for the merge and split steps (BI_{merge} and BI_{split}, respectively) represent the minimum BI value for a pair of components which presents satisfactory evidence of multi-modality in the respective step. Below the BI_{merge} threshold, the merge step will remove a component. Likewise, the split step will add a component only for values above

the $\mathrm{BI}_{\mathrm{split}}$) threshold. Specific values of the thresholds for the merge and split step were determined empirically by simulation in order to hold constant the false positive rate of detecting multi-modality near 0.10 for all sample sizes considered in the simulation study of the main text (50, 75, 100, and 500) as well as larger sample sizes (up to 1000). Parametric curves were fit to these values to allow for intermediate sample sizes. These curves, along with their values at the specific sample sizes used in this study, are illustrated in Figure S10; their formulas are given below.

$$\mathrm{BI}_{\mathrm{merge}} = \frac{1}{(1 + e^{-n/60+0.4})} + 1.2$$

$$\mathrm{BI}_{\mathrm{split}} = \frac{1}{(1 + e^{n/9-4.5})} + 3.4$$

Given that there was little change for both thresholds beyond sample sizes of 250-300, these curves level off beyond this range (as $n \to \infty$, $BI_{\mathrm{merge}} \to 2.2$, $BI_{\mathrm{split}} \to 3.4$).

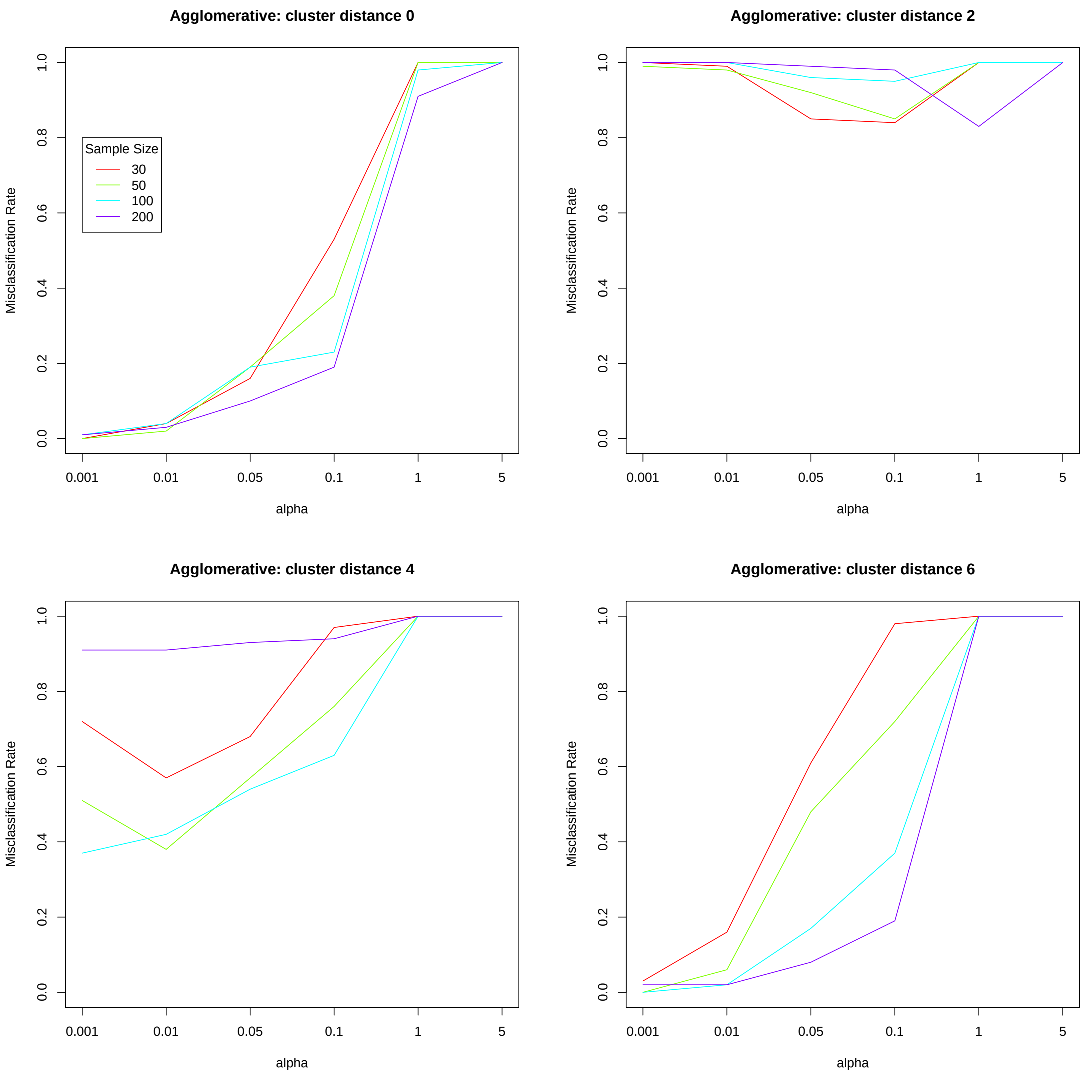

Figure S1: Proportion of replicates failing to identify the correct number of components in component distance scenario 1 (upper left), 2 (upper right), 3 (lower left) and 4 (lower right) using the agglomerative method

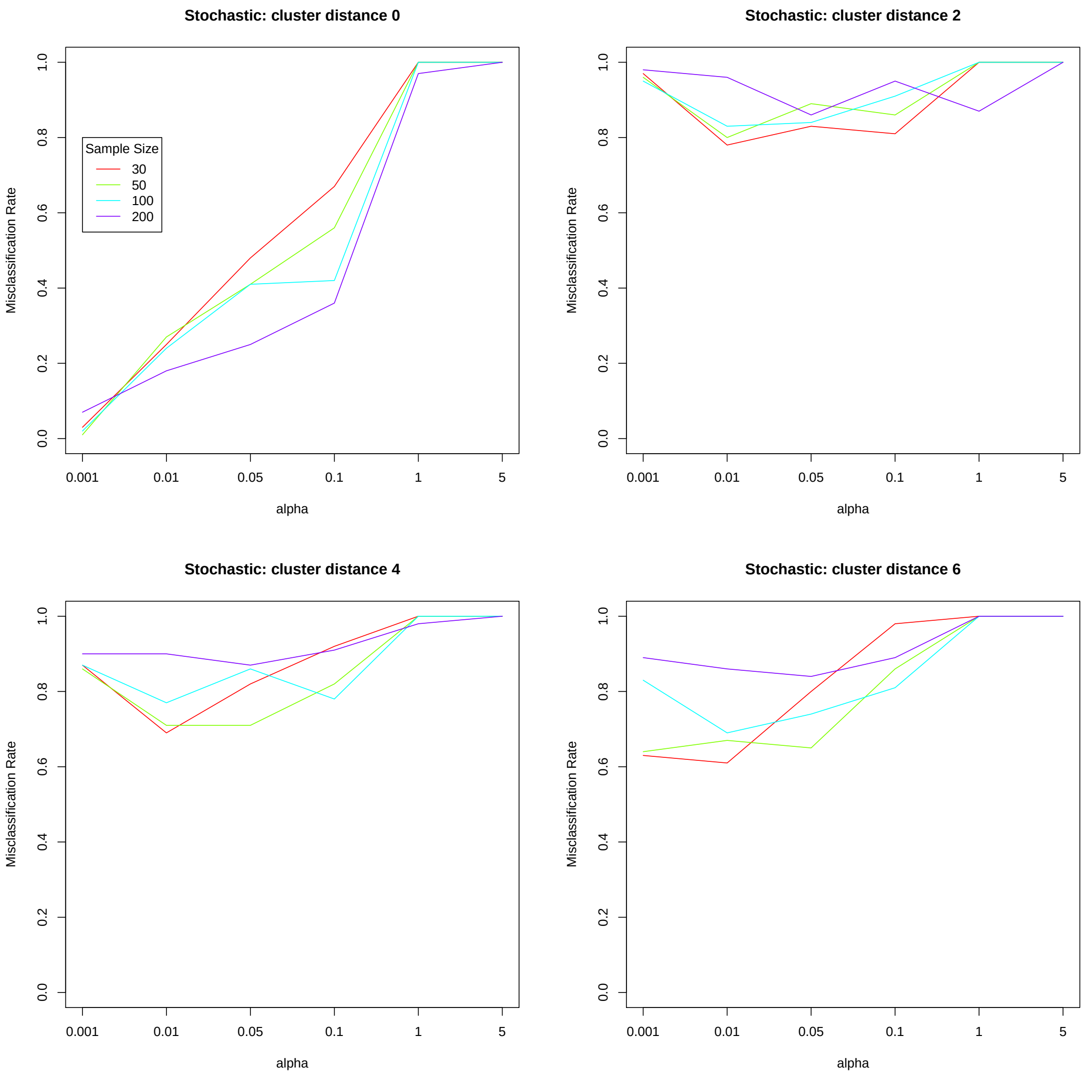

Figure S2: Proportion of replicates failing to identify the correct number of components in component distance scenario 1 (upper left), 2 (upper right), 3 (lower left) and 4 (lower right) using the stochastic method

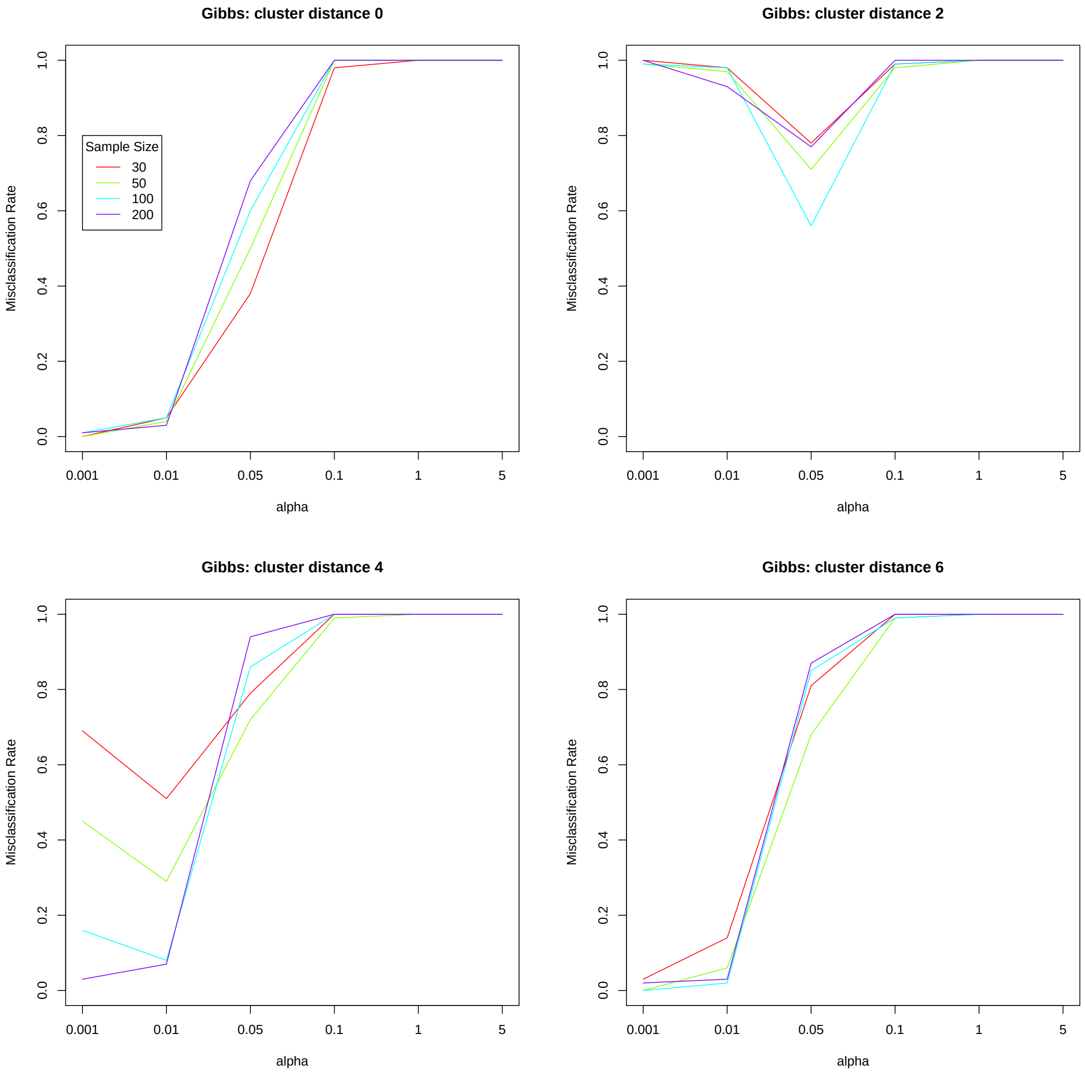

Figure S3: Proportion of replicates failing to identify the correct number of components in component distance scenario 1 (upper left), 2 (upper right), 3 (lower left) and 4 (lower right) using the Gibbs method

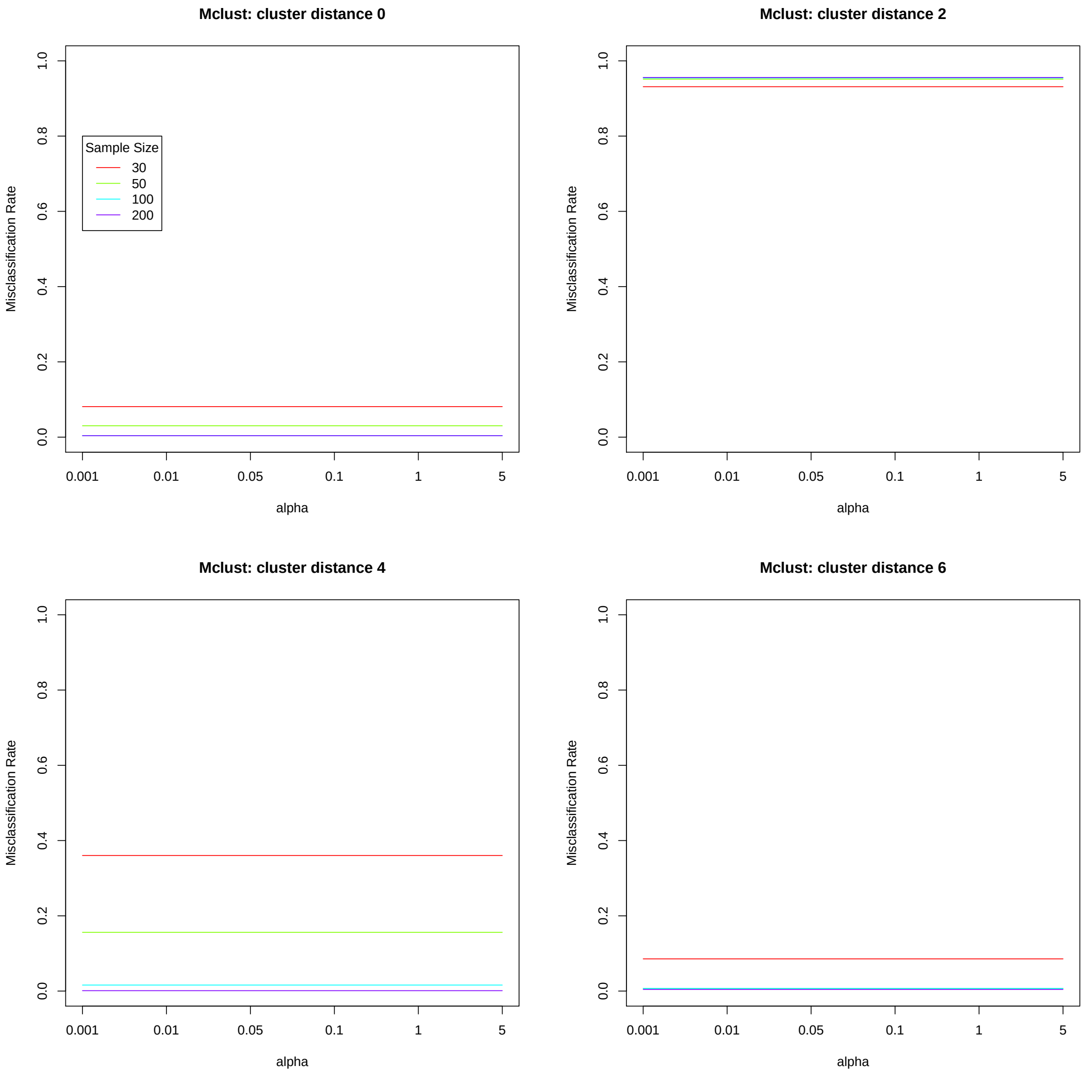

Figure S4: Proportion of replicates failing to identify the correct number of components in scenario 1 (upper left), 2 (upper right), 3 (lower left) and 4 (lower right) using Mclust

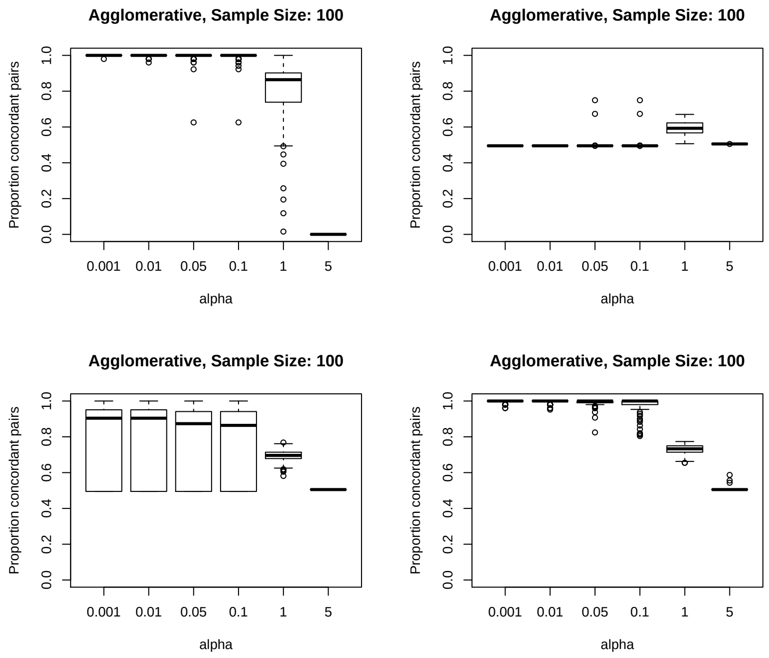

Figure S5: Rand Index for components identified in scenario 1 (upper left), 2 (upper right), 3 (lower left) and 4 (lower right) using the agglomerative method

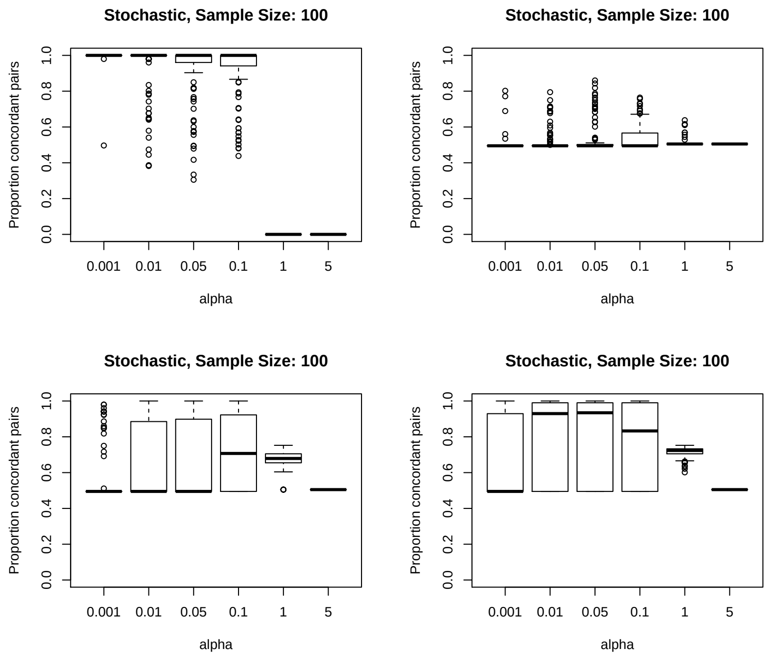

Figure S6: Rand Index for components identified in scenario 1 (upper left), 2 (upper right), 3 (lower left) and 4 (lower right) using the stochastic method

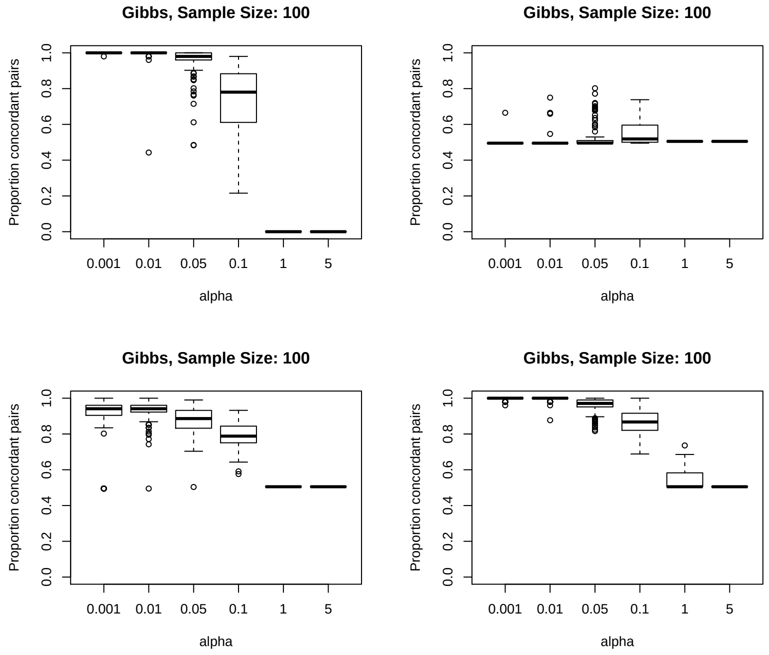

Figure S7: Rand Index for components identified in scenario 1 (upper left), 2 (upper right), 3 (lower left) and 4 (lower right) using the Gibbs method

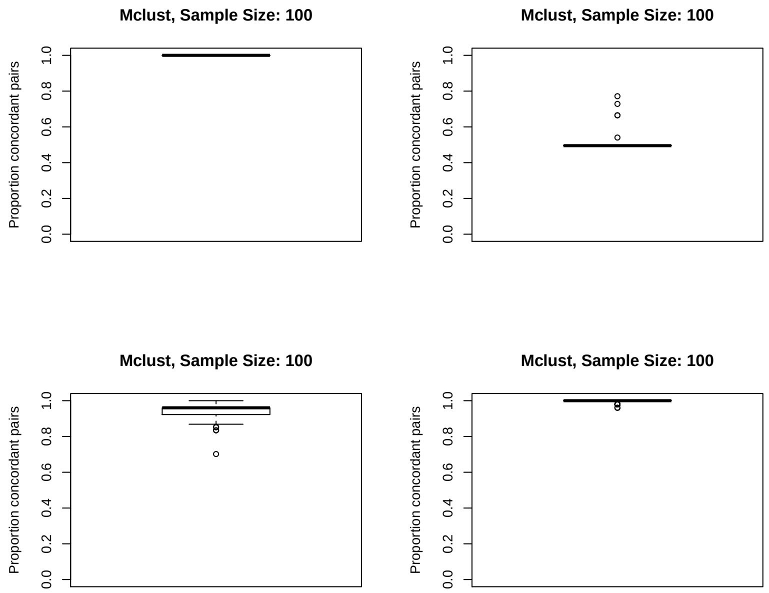

Figure S8: Rand Index for components identified in scenario 1 (upper left), 2 (upper right), 3 (lower left) and 4 (lower right) using Mclust

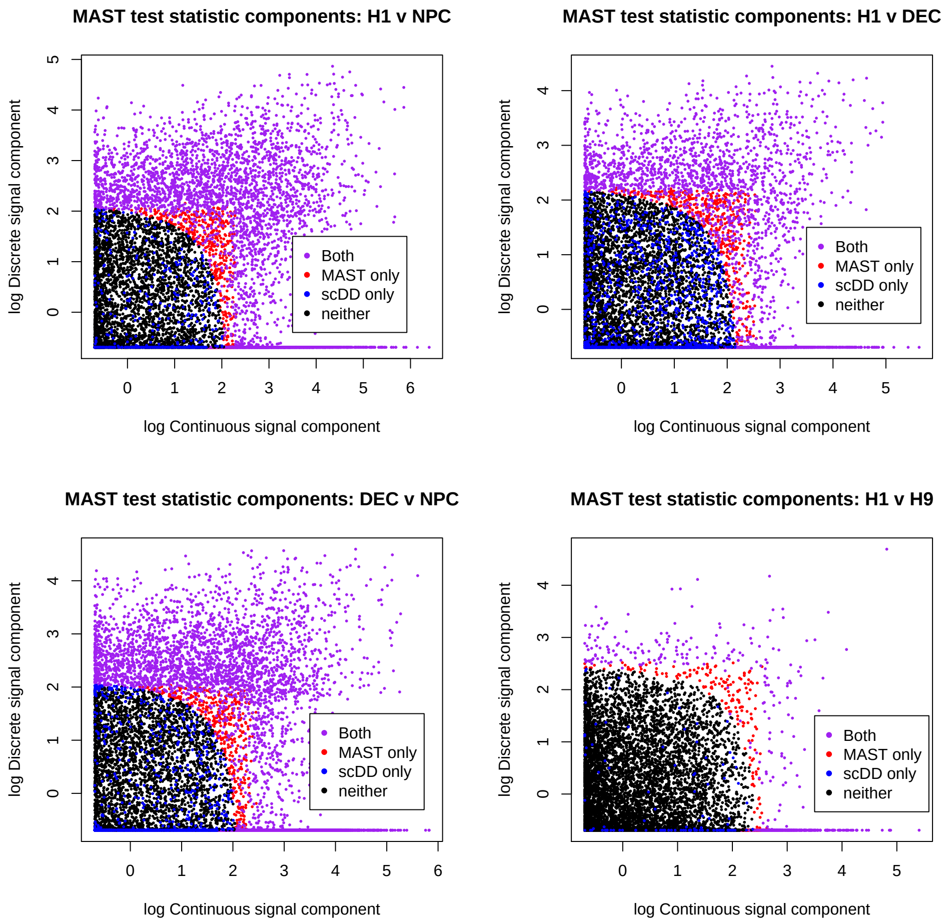

Figure S9: MAST test statistic components (continuous versus discrete) for comparison of H1 and NPC (upper left), H1 and DEC (upper right), NPC and DEC (lower left) and H1 and H9 (lower right), colored by whether or not the gene was differentially expressed by MAST and scDD.

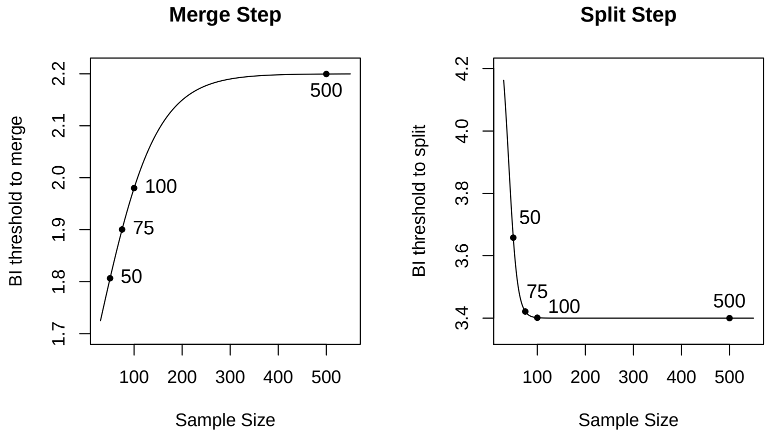

Figure S10: Bimodality Index (BI) thresholds used in the partition estimation step as a function of sample size. Left: In the merge step, a component is removed if any pair has a BI below the threshold. Right: In the split step, a component is added if as a result all pairs are separated by a BI above the threshold. Sample sizes used in the simulation study are highlighted.

References

C. E. Antoniak. Mixtures of Dirichlet processes with applications to bayesian nonparametric problems. *The annals of statistics*, pages 1152–1174, 1974.

C. A. Bush and S. N. MacEachern. A semiparametric Bayesian model for randomised block designs. *Biometrika*, 83(2):275–285, 1996.

C. Fraley, A. E. Raftery, T. B. Murphy, and L. Scrucca. MCLUST version 4 for r: Normal mixture modeling for model-based clustering, classification, and density estimation. Technical report, no. 597, Department of Statistics, University of Washington, 2012.

S. N. MacEachern. Estimating normal means with a conjugate style Dirichlet process prior. *Communications in Statistics-Simulation and Computation*, 23(3):727–741, 1994.

S. N. MacEachern and P. Müller. Estimating mixture of Dirichlet process models. *Journal of Computational and Graphical Statistics*, 7(2):223–238, 1998.

M. S. Shotwell. profdpm: An R package for MAP estimation in a class of conjugate product partition models. *Journal of Statistical Software*, 53(8):1–18, 2013.

M. S. Shotwell and E. H. Slate. Bayesian outlier detection with dirichlet process mixtures. *Bayesian Analysis*, 6(4):665–690, 2011.

C. A. Vallejos, S. Richardson, and J. C. Marioni. Beyond comparisons of means: understanding changes in gene expression at the single-cell level. *Genome biology*, 17(1):1, 2016.

J. Wang, S. Wen, W. F. Symmans, L. Pusztai, and K. R. Coombes. The bimodality index: a criterion for discovering and ranking bimodal signatures from cancer gene expression profiling data. *Cancer informatics*, 7:199, 2009.

L. Wang and D. B. Dunson. Fast bayesian inference in dirichlet process mixture models. *Journal of Computational and Graphical Statistics*, 20(1):196–216, 2011.

J. H. Ward Jr. Hierarchical grouping to optimize an objective function. *Journal of the American statistical association*, 58(301):236–244, 1963.
